# A multicenter survey of temporal changes in chemotherapy-induced hair loss in breast cancer patients

**DOI:** 10.1371/journal.pone.0208118

**Published:** 2019-01-09

**Authors:** Takanori Watanabe, Hiroshi Yagata, Mitsue Saito, Hiroko Okada, Tamiko Yajima, Nao Tamai, Yuko Yoshida, Tomoko Takayama, Hirohisa Imai, Keiko Nozawa, Takafumi Sangai, Akiyo Yoshimura, Yoshie Hasegawa, Takuhiro Yamaguchi, Kojiro Shimozuma, Yasuo Ohashi

**Affiliations:** 1 Department of Breast Cancer, National Hospital Organization Sendai Medical Center, Sendai, Japan; 2 Department of Breast care, Saitama Medical Center, Saitama Medical University, Kawagoe, Japan; 3 Department of Breast Oncology, Juntendo University, Tokyo, Japan; 4 Department of Health Communication, Graduate School of Medicine, The University of Tokyo, Tokyo, Japan; 5 EP-CRSU.Co.,Ltd. Clinical research promotion department, Tokyo, Japan; 6 Division of Health Sciences and Nursing, Graduate School of Medicine, The University of Tokyo, Tokyo, Japan; 7 Department of Breast Oncology, Juntendo University Hospital, Tokyo, Japan; 8 National Cancer Center, Center for Cancer Control and Information Services, Cancer Information Service Division, Tokyo, Japan; 9 Department of Medical and Pharmaceutical community healthcare, Graduate School of Medicine, The University of Tokyo, Tokyo, Japan; 10 National Cancer Center Hospital Appearance Support Center, Tokyo, Japan; 11 Department of General Surgery, Graduate School of Medicine, Chiba University, Chiba, Japan; 12 Department of Breast Oncology, Aichi Cancer Hospital, Nagoya, Japan; 13 Department of Breast Surgery, Hirosaki Municipal Hospital, Hirosaki, Japan; 14 Division of Biostatistics, Tohoku University Graduate School of Medicine, Sendai, Japan; 15 Department of Biomedical Sciences, College of Life Sciences, Ritsumeikan University, Kusatsu, Japan; 16 Department of Integrated Science and Engineering for Sustainable Society, Chuo University, Tokyo, Japan; University of Kentucky, UNITED STATES

## Abstract

**Purpose:**

Many breast cancer patients suffer from chemotherapy-induced hair loss. Accurate information about temporal changes in chemotherapy-induced hair loss is important for supporting patients scheduled to receive chemotherapy, because it helps them to prepare. However, accurate information, on issues such as the frequency of hair loss after chemotherapy, when regrowth starts, the condition of regrown hair, and the frequency of incomplete hair regrowth, is lacking. This study aimed to clarify the long-term temporal changes in chemotherapy-induced hair loss using patient-reported outcomes for chemotherapy-induced hair loss.

**Methods:**

We conducted a multicenter, cross-sectional questionnaire survey. Disease-free patients who had completed adjuvant chemotherapy consisting of anthracycline and/or taxanes for breast cancer within the prior 5 years were enrolled from 47 hospitals and clinics in Japan. Descriptive statistics were obtained in this study. The study is reported according to the STROBE criteria.

**Results:**

The response rate was 81.5% (1511/1853), yielding 1478 questionnaires. Hair loss occurred in 99.9% of patients. The mean time from chemotherapy until hair loss was 18.0 days. Regrowth of scalp hair occurred in 98% of patients. The mean time from the completion of chemotherapy to the beginning of regrowth was 3.3 months. Two years after chemotherapy completion, the scalp-hair recovery rate was <30% in approximately 4% of patients, and this rate showed no improvement 5 years after chemotherapy. Eighty-four percent of the patients initially used wigs, decreasing to 47% by 1 year after chemotherapy and 15.2% after 2 years. The mean period of wig use was 12.5 months. However, a few patients were still using wigs 5 years after completing chemotherapy.

**Conclusions:**

Our survey focused on chemotherapy-induced hair loss in breast cancer patients. We believe these results to be useful for patients scheduled to receive chemotherapy.

## Introduction

Recent advances in breast cancer therapies have improved the survival rate of patients. The 5-year relative survival rate has increased from 75% in 1975–1977 to 91% in 2006–2012 [1]. Chemotherapies have contributed immensely to this survival rate improvement [2]. The most common chemotherapy drugs for breast cancer include anthracyclines and taxanes [3]. The major side effects of these drugs are nausea, vomiting, and hair loss. Due to recent improvements in antiemetic therapy, nausea and vomiting have decreased [4,5]. However, the hair loss has not been ameliorated and many patients with breast cancer suffer hair loss due to chemotherapy, not only scalp hair loss, but also eyebrow and eyelash loss, as well as nail changes induced by the chemotherapy, which can be psychologically traumatic [6]. These side effects related to appearance issues diminish self-esteem and greatly reduce quality of life for patients [7].

In 2011, “The Management of Patient Appearance in Cancer Therapy Working Group” was established in Japan to consider the management of appearance issues affecting patients with breast cancer who receive chemotherapy. We began by investigating the past literature on temporal changes in chemotherapy-induced hair loss. Most patients who receive chemotherapy want to know what will happen in the future because such information allows them to prepare for anticipated changes. Therefore, information about issues such as the frequency of hair loss after chemotherapy, the time at which hair regrowth starts, the condition of the regrown hair, and the frequency of incomplete hair regrowth is useful for breast cancer patients. According to our literature search (search terms: "breast cancer" AND chemotherapy AND (alopecia OR "hair loss"); 828 papers (PubMed: from 1980 to 2013), there have been no comprehensive surveys focusing on these issues.

The main purpose of this study was to outline the long-term temporal changes in chemotherapy-induced hair loss using patient-reported outcomes pertaining to such hair loss. The data obtained in this study will be provided to breast cancer patients scheduled to receive chemotherapy.

## Materials and methods

We conducted a multicenter, cross-sectional questionnaire survey. Breast cancer patients who had completed adjuvant chemotherapy regimens were recruited from hospitals and clinics in Japan from April to October 2013. The following subjects were considered to meet the inclusion criteria; 1) Breast cancer patients who had completed adjuvant chemotherapy regimens containing anthracycline and/or taxanes within the prior 5 years, 2) Disease-free patients, 3) Patients who are living in Japan and able to understand Japanese, 4) Patients 20 years of age or older. There were no exclusion criteria.

The questionnaire was developed for this survey based on the opinions of members of our Working Group. We initially solicited questions from each member. Items related to scalp hair, head coverings, eyebrows / eyelashes, nails and the severity of side effects were collected. Regarding the severity of side effects, we chose items based on reports in the relevant literature [8–10]. After several meetings, we reached consensus and finalized the questionnaire (S1 Fig (Japanese version), S2 Fig (English version)). It contained 65 questions concerning the patients’ perceptions of physical and non-physical side effects (15 questions) and chemotherapy-induced hair loss or appearance issues (50 questions). The 50 questions concerning chemotherapy-induced hair loss or appearance issues were about scalp hair (13 questions), eyebrows (2), eyelashes (2), nails (4), equipment used such as wigs and caps (13), information acquisition (8), and the use of makeup (8). In this questionnaire, we asked about the conditions of scalp hair, eyebrows, eyelashes and nails at 3 different time points: before chemotherapy, 6 months after the beginning of scalp hair regrowth, and at the time of filling out the questionnaire survey.

At the outpatient clinic, the attending physician explained this research to the patients. They then filled out the first page of the questionnaire, writing the medicines that they had been administered. The patients were given the questionnaires and returned home with them. Only patients who agreed to participate in the study mailed the questionnaire directly to the data center after responding to the items surveyed. Descriptive statistics were obtained in this study. The Kaplan-Meier method was used to plot graphs of the wig usage period. Since some patients did not respond to all items, the total number of responses differed among items. In this study, the temporal changes in hair regrowth and nail condition were analyzed using patient groups classified according to number of years after completing chemotherapy.

Ethics approval was given by the ethics committee of Sendai Medical Center. Furthermore, the institutional review boards or the ethics committees of the 46 facilities participating in this survey (please see **Acknowledgments** below) approved this questionnaire survey. Since this trial did not use human biological specimens, written informed consent was not required according to the ethical guidelines for epidemiological research in Japan (Ministry of Health, Labour and Welfare, Japan. 2012) [11]. However, verbally-provided informed consent was required for this study. An explanatory document pertaining to this study was given to the patients with a brief explanation. Verbally-provided informed consent was documented in each patient's medical chart. Patients who did not agree to participate in the study did not mail in the questionnaire form. The study is reported according to the STROBE criteria [12].

## Results

### Descriptive summary of data

A total of 1511 patients from 47 hospitals and clinics returned the questionnaire to the data center, a response rate of 81.5% (1511/1853). Since 33 patients did not meet the inclusion criteria, the questionnaires returned by 1478 patients were analyzed. The mean age was 54.7 ± 10.4 (SD) years (range 17–79 years). Table 1 lists the characteristics of these patients. In 63.5% (938) of the patients, both anthracycline and taxanes were administered. In 20.2% (299) patients, only a taxane was administered, and in 16.3% (241), only anthracycline was administered. Approximately 70% of the patients received endocrine therapy. Since the duration of endocrine therapy exceeds 5 years, most of these patients were considered to have been receiving endocrine therapy at the time of filling out the questionnaire survey. The distribution of the patients by years from the completion of chemotherapy until participating in this survey was: < 1 year: 411 (27.8%), 1–2 years: 352 (23.8%), 2–3 years: 287 (19.4%), 3–4 years: 224 (15.2%), and 4–5 years: 204 (13.8%). Approximately 96% of the patients were treated and followed in cancer centers, university hospitals, or general hospitals.

**Table 1:** Patient characteristics (n = 1478).

		n			n			n
Age*			Hormone therapy**			Years after completing chemotherapy		
	≤39	101 (6.8%)		AI	494 (33.4%)		<1 year	411 (27.8%)
	40–49	372 (25.2%)		SERMs	348 (23.5%)		1–2 years	352 (23.8%)
	50–59	437 (29.6%)		LHRH-A+SERMs	112 (7.6%)		2–3 years	287 (19.4%)
	60–69	406 (27.5%)		SERMs+AI	50 (3.4%)		3–4 years	224 (15.2%)
	≥70	112 (7.6%)		LHRH-A	9 (0.6%)		4–5 years	204 (13.8%)
	No response	50 (3.4%)		LHRH-A+SERMs+AI	2 (0.1%)			
				LHRH-A+AI	1 (0.1%)	Institution		
Chemotherapy Drugs				None	462 (31.3%)		Cancer center	313 (21.2%)
	A+C+D	568 (38.4%)					University hospital	299 (20.2%)
	A+C+P	358 (24.2%)	Working status				General hospital	806 (54.5%)
	D	281 (19.0%)		Active worker	674 (45.6%)		Clinic	59 (4.0%)
	A+C	241 (16.3%)		Unemployed or Housework	793 (53.7%)		No response	1 (0.1%)
	P	17 (1.2%)		No response	11 (0.7%)			
	A+C+D+P	12 (0.8%)						
	P+D	1 (0.1%)						

*At time of survey.

** After chemotherapy. A: anthracycline, C: cyclophosphamide, D: docetaxel, P: paclitaxel, AI: aromatase inhibitors SERMs: selective estrogen receptor modulators, LHRH-A: luteinizing hormone-releasing hormone agonists

### Impact of side effects of chemotherapy

Among the 15 side effects that we had pre-selected for examination in this study, 82.6% of patients described hair loss as a severe or moderate side effect. When ranked by the total percentage of severe and moderate side effects, the most traumatic side effect was hair loss (Table 2). The second most traumatic side effect was fatigue (62.2%), while nail changes were in 7th place (50.7%) and nausea/vomiting (40.0%) in 10th place.

**Table 2:** Impact of side effects of chemotherapy.

	Symptoms*	Severe	Moderate	Mild	Very mild/None	No response
1	Hair loss	883 (59.7%)	339 (22.9%)	139 (9.4%)	97 (6.6%)	20 (1.4%).
2	Fatigue	461 (31.2%)	458 (31.0%)	301 (20.4%)	243 (16.4%)	15 (1.0%)
3	Length of treatment period	427 (28.9%)	457 (30.9%)	336 (22.7%)	235 (15.9%)	23 (1.6%)
4	Anxiety about the disease or treatment	473 (32.0%)	404 (27.3%)	288 (19.5%)	297 (20.1%)	16 (1.1%)
5	Medical expense	483 (32.7%)	393 (26.6%)	274 (18.5%)	312 (21.1%)	16 (1.1%)
6	Taste disorder	441 (29.8%)	334(22.6%)	260 (17.6%)	433 (29.3%)	10 (0.7%)
7	Nail change	381 (25.8%)	368 (24.9%)	320 (21.7%)	397 (26.9%)	12 (0.8%)
8	Burden on family	327 (22.1%)	354 (24.0%)	431 (29.2%)	340 (23.0%)	26 (1.8%)
9	Limb numbness	353 (23.9%)	309 (20.9%)	278 (18.8%)	521 (35.3%)	17 (1.2%)
10	Nausea/Vomiting	300 (20.3%)	291 (19.7%)	230 (15.6%)	641 (43.4%)	16 (1.1%)
11	Depressed mood	239 (16.2%)	352 (23.8%)	338 (22.9%)	528 (35.7%)	21 (1.4%)
12	Weight gain/Edema	281 (19.0%)	278 (18.8%)	264 (17.9%)	640 (43.3%)	15 (1.0%)
13	Frustration caused by not being able to a job or housework	242 (16.4%)	316 (21.4%)	356 (24.1%)	542 (36.7%)	22 (1.5%)
14	Discomfort during infusion	202 (13.7%)	326 (22.1%)	334 (22.6%)	598 (40.4%)	18 (1.2%)
15	Sleep disorder	204 (13.8%)	255 (17.3%)	310 (21.0%)	693 (46.9%)	16 (1.1%)

*Symptoms were ranked by the total percentage of severe and moderate side effects.

### Scalp hair

#### Hair loss

Among 1478 patients, 1476 provided answers about scalp hair loss. A total of 1474 (99.9%) of the 1476 patients had experienced hair loss. One patient responded that she had not experienced hair loss, and one patient answered that she did not remember. Regarding the degree of hair loss, 1463 patients provided questionnaire answers (Table 3). In 1385 (94.7%) patients, more than 80% of hair had been lost. The mean time from the initiation of chemotherapy until the start of hair loss was 18.0 ± 12.6 (SD) days. The data were essentially the same in each of the patient groups classified according to the number of years after completion of chemotherapy (<1 year: 18.9 days, 1–2 years: 18.2, 2–3 years: 18.1, 3–4 years: 15.5, 4–5 years: 18.0).

**Table 3:** Hair loss and nail changes during chemotherapy.

	Percentage of hair loss			
Hair	<30%	40–70%	>80%	total*
Scalp hair	28 (1.9%)	50 (3.4%)	1385 (94,7%)	1463
Eyebrows	306 (20.8%)	290 (19.7%)	873 (59.4%)	1469
Eyelashes	325 (22.2%)	257 (17.6%)	882 (60.2%)	1464
	Severity of nail changes			
Nails	Severe	Moderate	Mild	total*
Fingernails	688 (47.0%)	456 (31.2%)	319 (21.8%)	1463
Toenails	426 (29.7%)	487 (33.9%)	522 (36.4%)	1435

* Total number of patients who responded.

#### Regrowth of scalp hair

Regrowth of scalp hair occurred in 98% of the 1470 patients who responded to this question. The regrowth started during chemotherapy in 13% (193) of the patients, and after the completion of chemotherapy in 80% (1187). In patients whose regrowth started after chemotherapy, the mean time from the completion of chemotherapy to the beginning of regrowth was 3.3 ± 4.8 (SD) months. Thirty-one patients replied that regrowth did not occur. In 7 of these 31 patients, regrowth of scalp hair did not occur after more than 6 months had elapsed since the completion of chemotherapy (9, 21, 44, 45, 56, 58, and 60 months).

Table 4 shows the condition of the scalp hair 6 months after the beginning of regrowth. The scalp hair was thinner in 58% of the patients. Hair thickness was unchanged in 32%. Hair texture had become wavy or wavier in 63%. In 25% of the patients, hair texture was unchanged. Hair color was unchanged in 53% of patients, while in 38% the hair had become grayer or whiter.

**Table 4:** Condition of the scalp hair 6 months after the beginning of hair regrowth.

	Scalp hair condition	n
Thickness		
	Became thinner	793 (58.0%)
	No change	440 (32.2%)
	Became thicker	85 (6.2%)
	Others	50 (3.7%)
	Total*	1368
Texture		
	Became straight	19 (1.4%)
	No change	336 (25.0%)
	Became wavy/curly	398 (29.6%)
	Became wavier/curlier	448 (33.3%)
	Became less wavy/curly	59 (4.4%)
	Others	85 (6.3%)
	Total	1345
Color		
	Became dark	73 (5.3%)
	No change	717 (52.5%)
	Became grayer or whiter	525 (38.4%)
	Others	51 (3.7%)
	Total	1366

* Total number of patients who responded.

#### Scalp hair condition >6 months after the beginning of regrowth

The recovery rates by years after chemotherapy are shown in Table 5. This question was answered only by patients whose scalp hair regrowth had started >6 months prior to answering the questionnaire. In the group given chemotherapy <1 year prior to answering the questionnaire (<1 year group), 13.2% of patients replied that the hair recovery rate was <30%. In the 1–2 year group, the frequency of such patients decreased to 3.5%. Among the patients who had received chemotherapy >2 years earlier, the frequency did not decrease. These data suggest that the recovery process for hair loss stops at approximately 1 year after chemotherapy completion in most patients. For this reason, we used the data of the 1067 patients who had completed chemotherapy >1 year before responding to the questionnaire (>1 year group) in a subsequent analysis.

**Table 5:** Percentage of recovery by years after chemotherapy.

Patients per group		Percentage of recovery			
		<30%	40–70%	>80%	total*
Scalp hair recovery					
	<1 year group	29 (13.2%)	75 (34.1%)	116 (52.7%)	220**
	1–2 yeays group	12 (3.5%)	111 (32.5%)	219 (64.0%)	342
	2–3 years group	10 (3.5%)	96 (33.7%)	179 (62.8%)	285
	3–4 years group	8 (3.7%)	70 (32.0%)	141 (64.4%)	219
	4–5 years group	12 (6.0%)	71 (35.3%)	118 (58.7%)	201
Eyebrow recovery					
	<1 year group	58 (14.3%)	121 (29.7%)	228 (56.0%)	407
	1–2 yeays group	16 (4.7%)	89 (25.9%)	239 (69.5%)	344
	2–3 years group	13 (4.6%)	63 (22.4%)	205 (73.0%)	281
	3–4 years group	17 (7.8%)	52 (23.7%)	150 (68.5%)	219
	4–5 years group	15 (7.4%)	57 (28.2%)	130 (64.4%)	202
Eyelash recovery					
	<1 year group	64 (15.9%)	154 (38.2%)	185 (45.9%)	403
	1–2 yeays group	9 (2.6%)	107 (31.2%)	227 (66.2%)	343
	2–3 years group	12 (4.3%)	92 (33.0%)	175 (62.7%)	279
	3–4 years group	13 (6.0%)	64 (29.5%)	140 (64.5%)	217
	4–5 years group	15 (7.5%)	67 (33.5%)	118 (59.0%)	200
Fingernail recovery					
	<1 year group	69 (16.9%)	95 (23.3%)	244 (59.8%)	408
	1–2 yeays group	8 (2.3%)	43 (12.5%)	294 (85,2%)	345
	2–3 years group	1 (0.4%)	31 (11.0%)	250 (88.7%)	282
	3–4 years group	7 (3.2%)	20 (9.3%)	189 (87.5%)	216
	4–5 years group	3 (1.5%)	14 (7.1%)	182 (91.5%)	200
Toenail recovery					
	<1 year group	77 (16.9%)	118 (23.3%)	205 (59.8%)	400
	1–2 yeays group	14 (4.1%)	49 (14.4%)	277 (81.5%)	340
	2–3 years group	3 (1.1%)	35 (12.7%)	238 (86.2%)	276
	3–4 years group	12 (5.6%)	25 (11.7%)	177 (82.7%)	214
	4–5 years group	0 (0.0%)	23 (11.6%)	175 (88.4%)	198

* Total number of patients who responded.

** This question was asked only of patients whose scalp hair regrowth had started more than 6 months earlier.

A total of 417 of the 1067 patients (39%) responded that they had regions with poor scalp hair regrowth. The worst regrowth area was the forehead (77%, 322/417). The second was the parietal region (62%, 259/417), the third the occipital region (14%, 57/417). Among these patients, 30% replied that hair regrowth was poor in both the forehead and the parietal region, and 7% replied that hair regrowth was poor over the entire head.

#### Wigs

**Fig 1:**
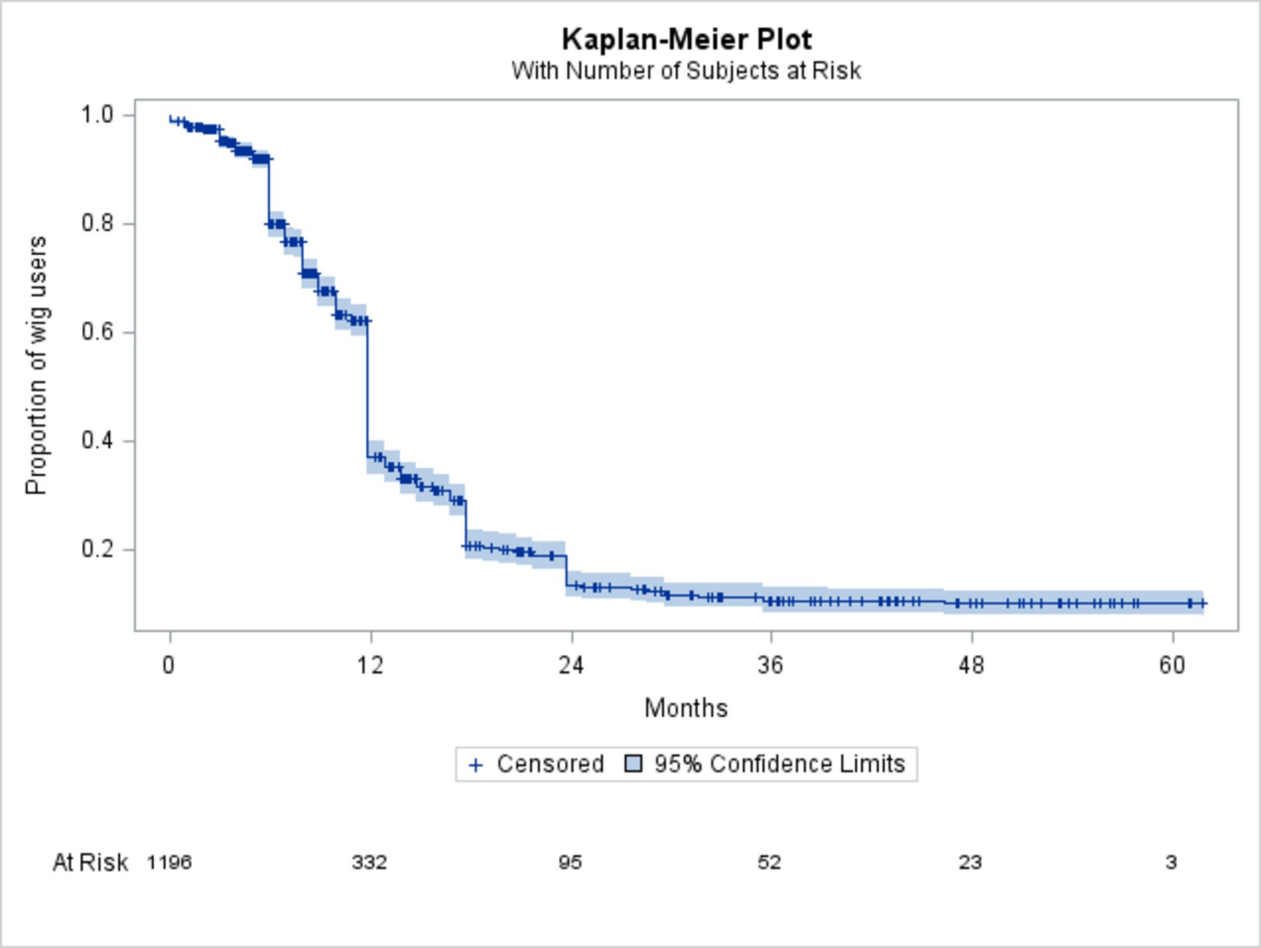
Period of wig use. The wig usage rates at 12, 24 and 36 months were estimated to be 37.3%, 13.5 and 10.3% based on the Kaplan-Meier plot.

During or after chemotherapy, 84% (1237/1478) of the patients used wigs. Regarding the wig use period, 1196 patients responded to this question. The mean period of wig use was 12.5 ± 9.7 (SD) months. However, a few patients were still using a wig more than 3 years after chemotherapy. Although this study was a cross-sectional study, temporal changes in the wig usage rate were analyzed by the Kaplan-Meier plot method using the responses collected. The Kaplan-Meier plot demonstrated wig usage rates at 12, 24 and 36 months of approximately 37%, 13% and 10%, respectively (Fig 1). Twenty-three patients in this study used wigs for more than 4 years after chemotherapy completion.

Approximately half of the patients who used wigs bought one wig. However, 25% of the patients bought two wigs and 14% three or more wigs.

### Eyebrows and eyelashes

Eyebrow hairs fell out in 90% of the 1478 patients and >80% eyebrow loss occurred in 59%. Eyelashes fell out in 88% and >80% of eyelash hairs were lost in 60% of patients (Table 3). The recovery rates by years after chemotherapy are shown in Table 4. In the <1 year group, the degree of recovery was obviously insufficient as compared to the other groups. These data also suggest that the process of recovery from the loss of eyebrows and eyelashes stops at approximately 1 year after chemotherapy in most patients. For the eyebrows and eyelashes, recovery >80% was seen in 60–70% of patients in the >1 year group.

### Nails

Severe or moderate fingernail changes occurred in 78% (1144/1463) and toenail changes in 64% (913/1435) of patients (Table 3). Specific fingernail changes were described by 988 patients. The total number of changes described was 1759. Color change (40%, 709/1759), deformation (20%, 352), brittleness (20%, 347), and lines on nails (14%, 249) were reported as major fingernail changes. Specific toenail changes were reported by 766 patients. The total number of changes described was 901. Color change (43%, 385/901), deformation (21%, 186), separation (13%, 120), lines on nails (7%, 61), and brittleness (6%, 56) were reported as major toenail changes.

The recovery rates by years after chemotherapy are shown in Table 4. Fingernail and toenail recovery rates >80% were seen in 80–90% of patients in the >1 year group.

## Discussion

Anthracycline and taxanes are the standard adjuvant chemotherapy agents for breast cancer [13–15]. Combinations of these two types of agents cause complete hair loss in most cases [16–18]. Many women report that it is more difficult to cope with hair loss than the loss of their breast because it is outwardly visible to others [19]. Hair loss is always rated highly when ranking the severity of chemotherapy side effects [8–10]. Furthermore, nausea and vomiting have recently decreased significantly. Thus, appearance issues have become relatively more important than they formerly were. Scalp cooling has been used to prevent chemotherapy-induced hair loss [20, 21]. Despite many years of clinical application and many related studies, the effectiveness of this intervention remains questionable [22]. In 2017, Nangia et al [23] reported that scalp cooling significantly reduced hair loss in breast cancer patients who received chemotherapy in a randomized clinical study. Although 100% of the patients who did not receive scalp cooling used wigs or head wraps, only 63% of those who received scalp cooling required wigs and wraps. However, in their study, the condition of scalp hair was observed after 4 cycles of taxane or anthracycline based chemotherapy. The condition after 8 cycles of combination chemotherapy with anthracycline and taxane-based treatment was not reported.

Our survey showed that 99.9% of patients with breast cancer have experienced hair loss due to anthracycline and/or taxane-containing chemotherapy. This suggests that all patients with breast cancer who will receive such chemotherapy should be encouraged to prepare for hair loss. This form of education helps the patient, first, to anticipate hair loss, realize its impact, verbalize an understanding of factors that cause hair loss, and learn self-care techniques [24,25]. Providing accurate expectations about the physical changes that occur during hair loss allows patients to prepare for both the psychological and the social changes associated with this side effect [26].

We believe that explaining a “typical” course of hair loss is useful for patients. The “typical” course would be as follows. Hair loss starts approximately 18 days after the first administration of chemotherapy and hair regrowth can be expected to begin 3 months after completing chemotherapy. However, the new scalp hair will likely be thinner and wavier for at least 6 months after the last course of chemotherapy. Hair color will not change in 60% of patients, while in 40% the hair will become grayer or whiter. Most patients use a wig for approximately 1 year after completing chemotherapy. Loss of eyebrow and eyelash hair during chemotherapy can be expected, and will exceed 80% after completion of chemotherapy. However, both will recover in 1 or 2 years. While this is the “typical” course, it is also important to inform patients that there are many variations. For example, the recovery of scalp hair might be delayed. A wig might be needed even 5 years after chemotherapy, though the probability of this is very low.

We used patient-reported outcomes in this study. Since these are subjective data, careful interpretation is necessary. For example, the mean time from chemotherapy until the start of scalp hair regrowth was 3.3 months in this survey. This meant that the patients noticed the regrowth of their scalp hair 3.3 months after the completion of chemotherapy. The timing of awareness of hair regrowth may depend on how carefully patients monitor their own conditions. Therefore, our present results may differ from strictly objective observations of hair regrowth. In this way, subjective information, as provided by patients, may differ from objective information. However, obtaining long-term objective information from many patients is highly laborious and costly. Considering that the purpose of this study was to "outline" long-term temporal changes, we feel that subjective information is clinically useful. Since this study included patients who received chemotherapy 1 to 5 years prior to being given the questionnaire, we must consider recall bias. However, for example, the reported mean times from chemotherapy until hair loss were similar regardless of the elapsed time since completion of chemotherapy. This observation suggests that although there are limitations, the quality of our data is acceptable.

Hair loss due to chemotherapy has been thought to, generally, be completely reversible. However, several authors have reported permanent or irreversible alopecia in patients with breast cancer after standard dose chemotherapy [27–30]. In most of these cases, docetaxel and anthracycline were administered. Although the incidence of permanent scalp alopecia is unknown, Kluger et al. roughly estimated that this side effect occurred in ≤2% of patients. Our data showed that approximately 4% of the patients who had completed chemotherapy >1 year before answering the questionnaire replied that their scalp hair recovery rate was <30%. This trend was essentially the same in patients who had received chemotherapy 2–5 years earlier. Furthermore, the frequency of patients whose scalp hair recovered by 40%-70% was >30% even 5 years after chemotherapy. In addition, this trend was also seen for eyelashes and eyebrows. Thus, many patients, more than expected, are suffer from appearance issues induced by chemotherapy. This suggests a need for long-term and careful support plans for these patients.

### Conclusion

This study was conducted to clarify the hair loss situation in patients with breast cancer after chemotherapy. It is important to provide the results of this study to both patients and medical practitioners. We hope that further detailed research will be performed and that studies focusing on support for patients and hair loss prevention will be carried out.

## Supporting information

S1 Data set Questionnaire data of 1478 patients. (XLSX)

S1 Fig Hair loss questionnaire (Japanese version). (PDF)

S2 Fig Hair loss questionnaire (English version). (PDF)
