# Supplementary material for: A multicenter survey of temporal changes in chemotherapy-induced hair loss in breast cancer patients
Source: PLoS One. 2019 Jan 9;14(1):e0208118. doi: 10.1371/journal.pone.0208118 (PMC6326423; doi:10.1371/journal.pone.0208118)

医師記入用

調査票をお渡しする患者さんの治療に関する情報について、下記にご記入ください。

1. 化学療法について

終了時期：20____年 ___月頃

使用薬剤に〇を付けて下さい	↓	サイクル数
**アンスラサイクリン系**		
アンスラサイクリン+シクロフォスファミド（+/- 5-FU）		
**タキサン系**		
毎週パクリタキセル		
3 週毎ドセタキセル		

＊ハーセプチンは記入不要です.

2. 内分泌療法について

使用薬剤に〇を付けて下さい ↓		開始時期	終了時期
使用なし			
LH-RH アゴニスト		20____年 ___月頃	継続中（　　）／20____年 __月頃
タモキシフェン		20____年 ___月頃	継続中（　　）／20____年 __月頃
アロマターゼ阻害剤		20____年 ___月頃	継続中（　　）／20____年 __月頃

3. 医療施設の種類：該当するものに丸をつけてください

（　　）がん専門病院
（　　）大学病院
（　　）一般総合病院
（　　）クリニック

患者さん用

アンケートは全部で 13 ページあります。
あなたにもっとも当てはまると思われるものを選んで回答してください。
答えたくない質問へは回答しなくてもけっこうです。
なお、結果は担当医へは公開されません。あなたの率直な意見をお答え下さい。

【アンケートの記入方法】

設問ごとに、あなたの考えに最も近いと思われる回答を選んで答えてください。

設問によっては、数字を〇で囲んで答えるもの、四角にチェックを入れて答えるものがあります。また、数字を記入するものや、あなたの考えを直接記入していただくものがあります。

複数回答を選んでもらいたい設問には、複数回答可という記載があります。

設問をよくお読みになり、最も適切な方法で回答してください。

(ア)　このアンケートを記入された日：

20____年____月____日

(イ)　あなたの生年月日：

19____年____月

(ウ)　あなたのお住まいはどちらですか。都道府県のみお答え下さい。

________________都道府県

(エ)　乳がんの手術を受けられた時期について教えてください。

（西暦）20____年____月頃

(オ)　現在、お仕事はなさっていますか

1）はい　→(オ)－1 へ　　2）いいえ　→(オ)－2 へ

<u>(オ)－1</u>. (オ)で「はい」と回答した方にうかがいます。どのような勤務形態で働いていますか。

1）フルタイム　2）パートタイム　3）その他（　　　　）

その仕事は乳がんの治療を受ける前と同じ仕事ですか

1)はい　2）いいえ

<u>(オ)－2</u>. (オ)で「いいえ」と回答した方にうかがいます。下記のいずれが当てはまりますか。

1）仕事をしていたが、退職した

2）休職中

3）元から仕事はしていない

(カ)　現在、どなたと同居されていますか（あてはまるものに○をつけてください）

1）夫　2）子　3）親　4）その他（　　　　）　5）独居

<u>生理の状況について伺います</u>

(キ)　この数ヶ月間に生理はありましたか。どちらかに○をつけてください。

1）はい　2）いいえ

(キ)で、「いいえ」と回答された方にうかがいます。生理はいつからありませんか。

1）化学療法を受けてから

2）化学療法を受ける前から→いつごろからですか。____年____月頃

<u>3）子宮または卵巣の手術を受けてから</u>：手術日；____年____月頃

→ 手術内容：(1) 子宮切除・(2) 卵巣切除（□片側・□両側・□不明）

4）その他（　　　　　　　　　　　　　）

01. 化学療法中に感じた苦痛についてうかがいます

化学療法中に苦痛に思ったことは何でしたか。それはあなたにとってどのくらい苦痛でしたか。1）〜15）のそれぞれについて、あなたの感じた強さにあてはまる数字を「0：全く苦痛に感じなかった」〜「4：非常に苦痛に感じた」の１つに〇をしてください。下記以外の項目が苦痛であった場合は、16）17）のその他の欄に具体的に記載して、苦痛の強さについてもご回答ください。

	全く苦痛に感じなかった	あまり苦痛に感じなかった	やや苦痛に感じた	ある程度苦痛に感じた	非常に苦痛に感じた
1） 吐き気・嘔吐	0	1	2	3	4
2） 脱毛	0	1	2	3	4
3） 全身倦怠感（だるさ）	0	1	2	3	4
4） 病状や治療への不安	0	1	2	3	4
5） 治療期間の長さ	0	1	2	3	4
6） 抑うつ感（気分の落ち込み）	0	1	2	3	4
7） 点滴の不快感	0	1	2	3	4
8） 家族に迷惑をかけること	0	1	2	3	4
9） 仕事や家事ができないことによるあせり	0	1	2	3	4
10） 睡眠障害	0	1	2	3	4
11） 味覚障害	0	1	2	3	4
12） 爪の変化	0	1	2	3	4
13） 体重増加・浮腫（むくみ）	0	1	2	3	4
14） 手足のしびれ	0	1	2	3	4
15） 治療にかかった費用	0	1	2	3	4
16） その他：（　　　　　　　　　）	0	1	2	3	4
17） その他：（　　　　　　　　　）	0	1	2	3	4

02. あなたの「頭髪」についてうかがいます

1. 化学療法を受ける前の頭髪の状況についてうかがいます。①～④それぞれについて、当てはまるところに○をつけてください。

①	量	1）少ない	2）多い	3）どちらでもない	
②	太さ	1）細い	2）太い	3）どちらでもない	
③	髪質	1）直毛	2）くせ毛	3）どちらでもない	
④	色	1）黒	2）白髪が多少混じっている	3）白髪が多い	4）その他

2. 抗がん剤（化学療法）を行っている間の頭髪の状況についてうかがいます。

① 治療中に脱毛は起こりましたか（あるいは、髪は抜けましたか）。（いずれかに○）

1）　はい → 脱毛が始まったのは、いつ頃からですか：治療開始後（　　）日頃
あるいは、（　　）週目頃
2）　いいえ
3）　わからない

② 抗がん剤治療中は、どのくらいの量の頭髪が抜けていたと思いますか。（1 つに○）

1）　ほとんど抜けなかった
2）　2、3 割程度抜けた
3）　半分くらい抜けた（4、5 割程度）
4）　半分より多く抜けた（6、7 割程度）
5）　ほぼ抜けた（8、9 割程度）
6）　すべて抜けた

3. 治療後、（現在までに）頭髪は生えましたか。（どちらかに○）
1）は　い → 4（次の質問）にお進みください。
2）いいえ → **【03.まゆ毛について】**（8 ページ）にお進みください。

4. 前の質問【3】で「はい」と回答した方にうかがいます。頭髪が（少しでも）生え始めた時期は、いつ頃ですか。（どちらかに○。カッコ内に数字）

1）治療終了後（　　）ヶ月頃もしくは（　　）年頃
2）治療中

5. 発毛後約半年間の頭髪の状況についてうかがいます。発毛後、治療前と比べて変化はありましたか。変化があった場合には、どのように変わりましたか。

（いずれかに〇）

(ア)　太さ　1）変化はなかった　　2）変化があった

↓ どのような変化がありましたか

①細くなった　②太くなった　③その他（具体的に記述してください　　　　　）

(イ)　髪質　1）変化はなかった　　2）変化があった

↓ どのような変化がありましたか

①直毛になった　②くせ毛になった　③くせ毛の程度が弱くなった　④くせ毛の程度が強くなった　⑤その他（具体的に記述してください　　　　　）

(ウ)　色　　1）変化はなかった　　2）変化があった

↓ どのような変化がありましたか

①黒くなった　②白髪が増えた　③その他（具体的に記述してください　　　　　）

以下の 6 から 8 の質問は、発毛後半年以上経過した方のみお答えください

6. 現在の頭髪の状況についてうかがいます。発毛後の最初の半年間と比べて変化はありましたか。変化があった場合には、どのように変わりましたか。（いずれかに〇）

(ア)　太さ　1）変化はなかった　　2）変化があった

↓ どのような変化がありましたか（複数選択可）

①細くなった ②太くなった ③治療前の太さに（戻った・戻ってきた）
④その他（具体的に記述してください　　　　　）

(イ)　髪質　1）変化はなかった　　2）変化があった

↓ どのような変化がありましたか（複数選択可）

①直毛になった ②くせ毛の程度が弱くなった　③くせ毛の程度が強くなった ④治療前の髪質に（戻った・戻ってきた）⑤その他（具体的に記述してください　　）

(ウ)　色　　1）変化はなかった　　2）変化があった

↓ どのような変化がありましたか（複数選択可）

①黒くなった ②白髪が増えた ③白髪が減った ④治療前の色に（戻った・戻ってきた） ⑤その他（具体的に記述してください　　　　）

7. 頭髪の量は、現在どのようになっていますか。（１つに〇）

1） 抗がん剤治療前より多くなった
2） 抗がん剤治療前と全く同じ状態になった（ほぼ 100%回復）
3） 抗がん剤治療前とほぼ同じ状態になった（8、9 割程度回復）
4） 大分戻った（6、7 割程度回復）
5） 少し戻った（4、5 割程度回復）
6） あまり戻っていない（2、3 割以下の回復）

8. 現在、頭髪が再生していない部分がありますか。ある場合は該当する部分を全て選んでください。

1）頭全体 2）前髪（前頭部） 3）頭の上部分（頭頂部） 4）耳の周り（側頭部）<br>5）頭の後ろ（後頭部） 6）うなじのあたり（後頚部）

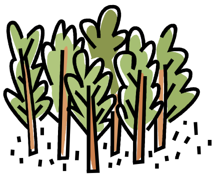

03. あなたの「まゆ毛」についてうかがいます

1. 抗がん剤治療中、どのくらいの量のまゆ毛が抜けたと思いますか。（１つに○）

1）ほとんど抜けなかった
2）2、3 割程度抜けた
3）半分くらい抜けた（4、5 割程度）
4）半分より多く抜けた（6、7 割程度）
5）ほぼ抜けた（8、9 割程度）
6）すべて抜けた

2. 現在、まゆ毛の量はどのようになっていますか。（１つに○）

1）抗がん剤治療前より多くなった
2）抗がん剤治療前と全く同じ状態になった（ほぼ 100%回復）
3）抗がん剤治療前とほぼ同じ状態になった（8、9 割程度回復）
4）大分戻った（6、7 割程度回復）
5）少し戻った（4、5 割程度回復）
6）あまり戻っていない（2、3 割以下の回復）

04. あなたの「まつ毛」についてうかがいます

1. 抗がん剤治療中、どのくらいの量のまつ毛が抜けたと思いますか。（１つに○）

1）ほとんど抜けなかった
2）2、3 割程度抜けた
3）半分くらい抜けた（4、5 割程度）
4）半分より多く抜けた（6、7 割程度）
5）ほぼ抜けた（8、9 割程度）
6）すべて抜けた

2. 現在、まつ毛の量はどのようになっていますか。（１つに○）

1）抗がん剤治療前より多くなった
2）抗がん剤治療前と全く同じ状態になった（ほぼ 100%回復）
3）抗がん剤治療前とほぼ同じ状態になった（8、9 割程度回復）
4）大分戻った（6、7 割程度回復）
5）少し戻った（4、5 割程度回復）
6）あまり戻っていない（2、3 割以下の回復）

05. あなたの「爪」についてうかがいます

1. 抗がん剤（化学療法）を行なっている間、あなたの「爪」は変わりましたか。

① 手の爪について　（１つに○）

1）ほとんど変わらなかった
2）半分くらい変わった
3）ほぼ変わった（8、9 割程度）

2）または 3）と回答した方にうかがいます。どのように変わりましたか。
具体的に記述してください。

（　　　　　　　　　　　　　　　　　　　　）

② 足の爪について　（１つに○）

1）ほとんど変わらなかった
2）半分くらい変わった
3）ほぼ変わった（8、9 割程度）

2）または 3）と回答した方にうかがいます。どのように変わりましたか。
具体的に記述してください。

（　　　　　　　　　　　　　　　　　　　　）

2. あなたの爪は、現在どのようになっていますか。

① 手の爪について（１つに○）

1）抗がん剤治療前と全く同じ状態になった（ほぼ 100％回復）
2）抗がん剤治療前とほぼ同じ状態になった（8、9 割程度回復）
3）大分戻った（6、7 割程度回復）
4）少し戻った（4、5 割程度回復）
5）わずかに戻った（2、3 割程度の回復）
6）あまり戻っていない（2、3 割以下の回復）

② 足の爪について（１つに○）

1）抗がん剤治療前と全く同じ状態になった（ほぼ 100％回復）
2）抗がん剤治療前とほぼ同じ状態になった（8、9 割程度回復）
3）大分戻った（6、7 割程度回復）
4）少し戻った（4、5 割程度回復）
5）わずかに戻った（2、3 割程度の回復）
6）あまり戻っていない（2、3 割以下の回復）

06.　脱毛、あるいは、頭髪の太さ・質感または色の変化があった方にうかがいます

1．以下の使用状況について教えてください。

①　ウィッグ　（１つに○）

使用頻度を教えてください
1）（ほぼ）毎日　2）数回/週　3）数回/月　4）使用しなかった

②　バンダナ　（１つに○）

使用頻度を教えてください
1）（ほぼ）毎日　2）数回/週　3）数回/月　4）使用しなかった

③　タオルキャップ　（１つに○）

使用頻度を教えてください
1）（ほぼ）毎日　2）数回/週　3）数回/月　4）使用しなかった

④　帽子　（１つに○）

使用頻度を教えてください
1）（ほぼ）毎日　2）数回/週　3）数回/月　4）使用しなかった

⑤　パーマ（１つに○。カッコ内に数字などを記入）

利用状況を教えてください
1）治療前よりも実施を控えた　→治療前は（　　）ヶ月おきに利用していた
2）治療終了後（　　）ヶ月頃もしくは（　　）年頃から再開した
3）治療前は利用していたが、現在利用していない
4）治療前から利用していなかった
5）その他（具体的に記述してください　　　　　　　　　　　　　　）

再開して、困ったこと，不便だったことはありましたか。具体的に教えてください。

（　　　　　　　　　　　　　　　　　　　　　　　　　　　　　　）

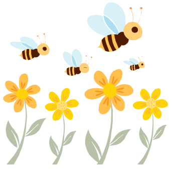

⑥　毛染め（1つに○。カッコ内に数字などを記入）

利用状況を教えてください
1）治療前よりも実施を控えた　→治療前は（　　）ヶ月おきに利用していた
2）治療終了後（　　）ヶ月頃もしくは（　　）年頃から再開した
3）治療前は利用していたが、現在利用していない
4）治療前から利用していなかった
5）その他（具体的に記述してください　　　　　　　　　）

再開して、困ったこと、不便だったことはありましたか。具体的に教えて下さい。

（　　　　　　　　　　　　　　　　　　　　　　　　）

⑦　①～⑥の他に利用していたものはありますか。（どちらかに○）

1）なかった　2）あった

2）と回答された方にうかがいます。具体的に教えて下さい。

（　　　　　　　　　　　　　　　　　　　　　　　　）

⑧　他にどのようなものがあったら良かったと思いますか。

（　　　　　　　　　　　　　　　　　　　　　　　　）

2.　ウィッグを使用されていた方にうかがいます

①　いくつ購入しましたか。（複数回答可）

1）1ヶ
2）2ヶ
3）3ヶ
4）4ヶ以上
5）購入したがウィッグを使用しなかった
6）購入せずにウィッグをレンタルした

②　価格帯はどのくらいのものを選びましたか（複数回答可）。1個あたりの価格を教えて下さい。

1）5万円未満　2）5万円～10万円未満　3）10万円以上

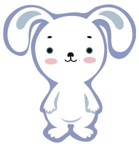

③ ウィッグの選び方について困ったことはありますか。（どちらかに○）

1）なかった　2）あった

2）と回答された方にうかがいます。どのような点で困りましたか。具体的に記述して下さい。

（　　　　　　　　　　　　　　　　　　　　　）

④ ウィッグの使い方について困ったことはありますか。（どちらかに○）

1）なかった　2）あった

2）と回答された方にうかがいます。どのような点で困りましたか。具体的に記述して下さい。

（　　　　　　　　　　　　　　　　　　　　　）

⑤ 化学療法終了後、どれくらいの期間、ウィッグを使用されていましたか。（どちらかに○をつけてください。1）の場合は、カッコに数字を記入してください）

1）約（　）年（　）ヶ月使用した<br>2）現在も使用している

07. ここからは、あなたが実際に受けられた頭髪、まゆ毛、まつ毛、爪に関する情報や支援についてうかがいます。あなたご自身が受けられたもの、経験されたものがありましたら、教えてください

1. 頭髪、まゆ毛、まつ毛、爪に関し、何らかの説明や情報を入手することはできましたか。（どちらかに○）

1) はい　　→ ①（次の質問）にお進みください
2) いいえ　→　2（14 ページ）にお進みください

① 「はい」と回答した方にうかがいます。情報の入手先は、どこですか。あてはまるものすべてに□にチェック（レ）を入れてください。

□医師①　□看護師②　□薬剤師③　□相談支援センター相談員④<br>□脱毛に関する専門スタッフ⑤　□ボランティア⑥　□家族⑦　□友人⑧　□知人⑨<br>□がんの経験者⑩　□インターネット⑪　□本⑫　□美容室⑬<br>□その他（具体的に：　　　　　　）⑭

② 頭髪に関する情報について、その説明や情報は、あなたにとって十分でしたか。
（１つに〇）

1）十分であった　2）ほぼ十分であった　3）まあまあ十分であった<br>4）あまり十分でなかった　5）全く十分でなかった

1）または 2）と回答された方にうかがいます。どのような情報が役に立ちましたか。
具体的に教えてください。

（　　　　　　　　　　　　　　　　　　　　）

4）または 5）と回答された方にうかがいます。十分でなかった理由を教えてください。

（　　　　　　　　　　　　　　　　　　　　）

③ まゆ毛に関する情報について、その説明や情報は、あなたにとって十分でしたか。
（１つに〇）

1）十分であった　2）ほぼ十分であった　3）まあまあ十分であった<br>4）あまり十分でなかった　5）全く十分でなかった

1）または 2）と回答された方にうかがいます。どのような情報が役に立ちましたか。
具体的に教えてください。

（　　　　　　　　　　　　　　　　　　　　）

4）または 5）と回答された方にうかがいます。十分でなかった理由を教えてください。

（　　　　　　　　　　　　　　　　　　　　）

④ まつ毛に関する情報について、その説明や情報は、あなたにとって十分でしたか。
（１つに〇）

1）十分であった　2）ほぼ十分であった　3）まあまあ十分であった<br>4）あまり十分でなかった　5）全く十分でなかった

1）または 2）と回答された方にうかがいます。どのような情報が役に立ちましたか。
具体的に教えてください。

（　　　　　　　　　　　　　　　　　　　　）

4）または 5）と回答された方にうかがいます。十分でなかった理由を教えてください。

（　　　　　　　　　　　　　　　　　　　　）

⑤ 爪に関する情報について、その説明や情報は、あなたにとって十分でしたか。
（１つに○）

1）十分であった　2）ほぼ十分であった　3）まあまあ十分であった<br>4）あまり十分でなかった　5）全く十分でなかった

1）または 2）と回答された方にうかがいます。どのような情報が役に立ちましたか。
具体的に教えてください。

（　　　　　　　　　　　　　　　　　　　　　　　　　　　　　　）

4）または 5）と回答された方にうかがいます。十分でなかった理由を教えてください。

（　　　　　　　　　　　　　　　　　　　　　　　　　　　　　　）

⑥ 情報提供や支援はいつ受けられましたか（複数回答可）

1） 抗がん剤治療を受ける前<br>2） 抗がん剤治療を受けている最中<br>3） 抗がん剤治療終了後

2. 治療後にあった方がよかった情報があればご記入ください。

（　　　　　　　　　　　　　　　　　　　　　　　　　　　　　　）

3. その他、脱毛に関することでお気づきのことがあれば、ご記入ください。

（　　　　　　　　　　　　　　　　　　　　　　　　　　　　　　）

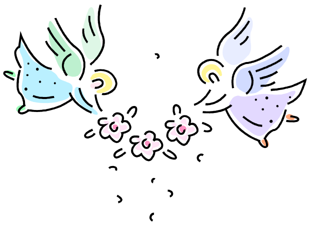

08. 最後に、治療前および現在のあなたのメイクの内容や仕方についてうかがいます。
それぞれ 1）～ 3）のいずれかに〇をつけてください

(ア)化学療法前からまゆ毛は描いていましたか・・ 1）はい　2）いいえ

(イ)化学療法中まゆ毛を描きましたか・・ 1）はい　2）いいえ

(ウ)化学療法と関係なくまゆ毛を描きたいと思いますか・・ 1）はい　2）いいえ

(エ)アートメイク(マイクロピグメンテーション)はご存じでしたか・・ 1）はい　2）いいえ

(オ)化学療法直前または化学療法中にアートメイク（マイクロピグメンテーション）を利用されましたか・・ 1）はい　2）いいえ

＊はいと答えた方にうかがいます

① アートメイクを利用して良かったと思いますか・・ 1）とても　2）まあまあ　3）いいえ

② 化学療法と関係なくアートメイクを利用したいと思いますか・・ 1）はい　2）いいえ

(カ)化学療法中につけまつ毛を利用されたことはありますか・・ 1）はい　2）いいえ

＊はいと答えた方にうかがいます

① つけまつ毛は役に立ちましたか・・ 1）とても　2）まあまあ　3）いいえ

② つけまつげの使用頻度は・・ 1）たまに　2）ときどき　3）ほぼ毎日

(キ)化学療法中に、爪のケアに関して何か工夫をされましたか・・ 1）はい　2）いいえ

＊はいと答えた方にうかがいます

① 爪の変化を隠すためにマニキュアを利用した・・ 1）はい　2）いいえ

② 爪を保護するためにジェルネイルを利用した・・ 1）はい　2）いいえ

③ 爪の切り方を工夫した・・ 1）はい　2）いいえ

④ 爪に関して，他の工夫をした方は、カッコ（　）内に具体的にお書きください

（　　　　　　　　　　　　　　　　　　　　）

(ク)爪のケアに関して、他にどのようなものがあったら良かったと思いますか。

（　　　　　　　　　　　　　　　　　　　　）

以上で、アンケートはすべて終わりです。
ご協力ありがとうございました。

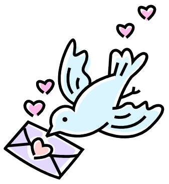

Supplement: S1 Fig — (PDF) [file pone.0208118.s002.pdf]
