# Supplementary material for: A multicenter survey of temporal changes in chemotherapy-induced hair loss in breast cancer patients
Source: PLoS One. 2019 Jan 9;14(1):e0208118. doi: 10.1371/journal.pone.0208118 (PMC6326423; doi:10.1371/journal.pone.0208118)

For physicians

Please fill the information pertaining to patient treatment.

1. Chemotherapy

Time of completion of therapy: around [month] ___, [year] 20____

Please circle the administered drug. ↓		Cycle number
**Anthracycline**		
Anthracycline + cyclophosphamide (+/- 5-FU)		
**Taxane**		
Paclitaxel, every week		
Docetaxel, every 3 weeks		

* HERCEPTIN does not have to be reported.

2. Endocrine therapy

Please circle the administered drug. ↓		Time of initiation	Time of completion
No drug administered			
LH-RH agonist		Around [month] ___, [year] 20____	Ongoing ( ) / around [month] ___, [year] 20___
Tamoxifen		Around [month] ___, [year] 20____	Ongoing ( ) / around [month] ___, [year] 20___
Aromatase inhibitor		Around [month] ___, [year] 20____	Ongoing ( ) / around [month] ___, [year] 20___

3. Type of medical institution: please check the appropriate selection.

( ) Specialized cancer hospital
( ) University hospital
( ) General hospital
( ) Clinic

For patients

This questionnaire consists of a total of 14 pages in total.
Please choose the most appropriate response from among the options provided to answer the question.
You do not have to answer particular questions if you do not want to.
Your results will not be disclosed to your doctor. Please let us know your true feelings.

【How to complete the questionnaire】

For each question, please choose the answer that seems to best describe your feeling.

Depending on the question, you may have to answer by circling the appropriate number or by checking the appropriate box. Some questions also ask you to write numbers, and others ask you to describe your feeling directly.

In the event of multiple answers being possible, the instructions will read, "Multiple answers allowed."

Please read the questions carefully and answer in the most appropriate way.

A) Date of completing this questionnaire: [month]____[day]____, [year] 20___

B) Your date of birth: [month]____, [year]19___

C) Where do you live? Please provide only the name of the state where you live.

[name of state]_______________

D) Please let us know when you underwent surgery for breast cancer.

Around [month]____, [year (western calendar)] 20___

E) Do you have a job at present?

1) Yes → Go to question E)-1. 2) No → Go to question E)-2.

E) -1. This question is for patients who answered “Yes” to question E). In what capacity do you work?

1) Full-time 2) Part-time 3) Others ( )

Is it the same job as you had before receiving treatment for breast cancer?

1) Yes 2) No

E) -2. This question is for patients who answered “No” to question E). Which of the following descriptions appy to you?

1) I used to have a job, but I retired.

2) I am on leave from my job.

3) I have never had a job.

F) With whom do you live at present? (Please circle the appropriate answers.)

1) Husband 2) Children 3) Parents 4) Others ( ) 5) Alone

Concerning your menstruation

G) Have you had your period in recent months? Please circle one of the following.

1) Yes 2) No

This question is for patients who answered “No” to the question G). When did your periods stop?

1) At the time of starting chemotherapy.

2) Before chemotherapy. → When? Around [month]____, [year] ____

3) At the time of surgery on the uterus or the ovary: date of surgery; around [month]____, [year] ____

→ Details of surgery: (1) hysterectomy • (2) ovariectomy (□ Unilateral • □ Bilateral • □ Unknown)

4) Others ( )

01. Concerning the events you experienced during chemotherapy.

Did you suffer any of the following events during chemotherapy? What was the severity? For items 1) to 15) below, please circle one of the following numbers “0: No suffering” to “4: Severe suffering” to show the extent to which you were adversely affected.

If you suffered any events other than the following items, please describe them and their severity in columns 16) or 17).

	No suffering	Less suffering	Slight suffering	Some degree of suffering	Severe suffering
1） Nausea • vomiting	0	1	2	3	4
2） Hair loss	0	1	2	3	4
3） General malaise (listlessness)	0	1	2	3	4
4） Anxiety about the disease or treatment	0	1	2	3	4
5） Length of treatment period	0	1	2	3	4
6） Depressed mood (depression)	0	1	2	3	4
7） Discomfort at infusion	0	1	2	3	4
8） Bothering family	0	1	2	3	4
9） Impatience caused by not being able to do a job or housework	0	1	2	3	4
10） Sleep disorder	0	1	2	3	4
11） Taste disorder	0	1	2	3	4
12） Nail change	0	1	2	3	4
13） Weight gain • edema (swelling)	0	1	2	3	4
14） Numbness of a limb	0	1	2	3	4
15） Medical expense	0	1	2	3	4
16） Others: ( )	0	1	2	3	4
17） Others: ( )	0	1	2	3	4

02. Concerning your "scalp hair"

1. Concerning the condition of your scalp hair <u>before receiving chemotherapy</u>. For items ① to ④ below, please circle the appropriate answer.

①	Amount	1) Scarce	2) Abundant	3) Neither		
②	Thickness	1) Thin	2) Thick	3) Neither		
③	Texture	1) Straight	2) Wavy or curly	3) Neither		
④	Color	1) Dark	2) Gray		3) White	4) Others

2. Concerning the condition of your scalp hair <u>while receiving anticancer agent(s) (chemotherapy)</u>

① Did you experience hair loss (or did your hair fall out) during treatment? (Circle one of the following.)

1） Yes → When did the hair loss start? : About (　　) days after the beginning of treatment,
or about (　　) weeks after the beginning of treatment
2） No
3） Cannot remember

② How much of your hair seemed to fall out during treatment with the anticancer agent(s)? (Circle the appropriate answer.)

1） Little
2） 20% - 30%
3） About half (40% -50%)
4） More than half (60% - 70%)
5） Almost all (80% - 90%)
6） All

3. Has your hair grown back (after treatment) yet? (Circle one of the following.)

1） Yes → Please go to question 4.
2） No → Please go to【**03. Eyebrows**】(page 9).

4. This question is for patients who answered "Yes" to the previous question【3】. When did your hair start re-growing (even if slightly)? (Circle one of the following. Write the number in the blank.)

1） About (　　) months or (　　) years after the completion of treatment
2） During treatment

5. Concerning the condition of your scalp hair after 6 months of hair regrowth

Did you experience any changes in your regrown hair as compared to before treatment? If so, how did your hair change? (Circle one of the following.)

A) Thickness 1) No change 2) Changed

⇩ How did it change?

① Became thinner ② Became thicker ③ Others (Please describe in detail.)

B) Texture 1) No change 2) Changed

⇩ How did it change?

① Became straight ② Became wavy/curly ③ Became less wavy/curly ④ Became more wavy/curly ⑤ Others (Please describe in detail.)

C) Color 1) No change 2) Changed

⇩ How did it change?

① Became dark ② Became more gray or white ③ Others (Please describe in detail.)

Please answer Questions 6 to 8 below only if more than 6 months have passed since hair regrowth.

6. Concerning the current condition of your scalp hair. Has it changed as compared to the first 6 months after hair regrowth? If so, how has it changed? (Circle one of the following.)

A) Thickness 1) No change 2) Changed

⇩ How has it changed? (Multiple answers allowed)

① Became thinner ② Became thicker ③ (Became・Becoming) as thick as before treatment ④ Others (Please describe in detail.)

B) Texture 1) No change 2) Changed

⇩ How has it changed? (Multiple answers allowed)

① Became straight ② Became less wavy/curly ③ Became more wavy/curly ④ (Became • Becoming) the same texture as before treatment ⑤ Others (Please describe in detail.)

C) Color 1) No change 2) Changed

⇩ How has it changed? (Multiple answers allowed)

① Became dark ② Became more gray or white ③ Became less gray ④ (Became • Becoming) the same color as before treatment ⑤ Others (Please describe in detail.)

7. How thick is your scalp hair at present? (Circle the appropriate answer.)

1） Thicker than before treatment with anticancer agent(s)

2） Exactly the same condition as before treatment with anticancer agent(s) (almost 100% recovery)

3） Almost the same condition as before treatment with anticancer agent(s) (80% - 90% recovery)

4） A lot like before treatment (60% - 70% recovery)

5） A little like before treatment (40% - 50% recovery)

6） Not much like before treatment (below 30% recovery)

8. Are there currently any parts of your scalp where hair has not regrown? If yes, please choose all that apply.

1) The entire head 2) Bangs (the forehead) 3) Top of the head (the parietal region) 4) Around the ears (the temporal region) 5) Back of the head (the occipital region) 6) Around the nape of the neck (the posterior region of the neck)

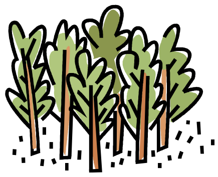

03. Concerning your "eyebrows"

1. How much of your eyebrow hair seemed to fall out during treatment with the anticancer agent(s)? (Circle the appropriate answer.)

1） Little
2） 20% - 30%
3） About half (40% - 50%)
4） More than half (60% - 70%)
5） Almost all (80% - 90%)
6） All

2. How thick are your eyebrows at present? (Circle the appropriate answer.)

1） Thicker than before treatment with anticancer agent(s)
2） Exactly the same condition as before treatment with anticancer agent(s) (almost 100% recovery)
3） Almost the same condition as before treatment with anticancer agent(s) (80% - 90% recovery)
4） A lot like before treatment (60% - 70% recovery)
5） A little like before treatment (40% - 50% recovery)
6） Not much like before treatment (below 30% recovery)

04. Concerning your "eyelashes"

1. How much of your eyelashes seemed to fall out during treatment with the anticancer agent(s)? (Circle the appropriate answer.)

1） Little
2） 20% - 30%
3） About half (40% - 50%)
4） More than half (60% - 70%)
5） Almost all (80% - 90%)
6） All

2. How thick are your eyelashes at present? (Circle the appropriate answer.)

1） Thicker than before treatment with anticancer agent(s)
2） Exactly the same condition as before treatment with anticancer agent(s) (almost 100% recovery)
3） Almost the same condition as before treatment with anticancer agent(s) (80% - 90% recovery)
4） A lot like before treatment (60% - 70% recovery)
5） A little like before treatment (40% - 50% recovery)
6） Not much like before treatment (below 30% recovery)

05. Concerning your "nails"

1. Did your "nails" change during treatment with the anticancer agent(s) (chemotherapy)?

① Hand nails (Circle the appropriate answer.)

1） Minimally changed
2） Moderately changed
3） Much changed (80% - 90%)

This question is for patients who chose answer 2) or 3) to the above question. How did your nails change? Please describe in detail.

(   )

② Toe nails (Circle the appropriate answer.)

1） Minimally changed
2） Moderately changed
3） Much changed (80% - 90%)

This question is for patients who chose answer 2) or 3) to the above question. How did your nails change? Please describe in detail.

(   )

2. What is the condition of your nails at present?

① Hand nails (Circle the appropriate answer.)

1） Exactly the same condition as before treatment with the anticancer agent(s) (almost 100% recovery)
2） Almost the same condition as before treatment with the anticancer agent(s) (80% - 90% recovery)
3） A lot like before treatment (60% - 70% recovery)
4） A little like before treatment (40% - 50% recovery)
5） Slightly like before treatment (20% - 30% recovery)
6） Not much like before treatment (below 20% recovery)

② Toe nails (Circle the appropriate answer.)

1） Exactly the same condition as before treatment with anticancer agent (almost 100% recovery)
2） Almost the same condition as before treatment with anticancer agent (80% - 90% recovery)
3） A lot like before treatment (60% - 70% recovery)
4） A little like before treatment (40% - 50% recovery)
5） Slightly like before treatment (20% - 30% recovery)
6） Not much like before treatment (below 20% recovery)

06. To patients who experienced hair loss, or changes in the thickness, texture or color of their hair

1. Concerning usage of the following.

① Wig (Circle the appropriate answer.)

Concerning the frequency of use.

1) (almost) every day 2) several times/week 3) several times/month 4) No use

② Bandana (Circle the appropriate answer.)

Concerning the frequency of use.

1) (almost) every day 2) several times/week 3) several times/month 4) No use

③ Towel cap (Circle the appropriate answer.)

Concerning the frequency of use.

1) (almost) every day 2) several times/week 3) several times/month 4) No use

④ Hat (Circle the appropriate answer.)

Concerning the frequency of use.

1) (almost) every day 2) several times/week 3) several times/month 4) No use

⑤ Perms (Circle the appropriate answer. Write numbers etc. in the blanks.)

Concerning the usage conditions.

1) Refrained from having perms as compared to before treatment → I used to have a perm every ( ) months before treatment.

2) Started having perms again about ( ) months or ( ) years after the completion of treatment.

3) I had perms before treatment, but I do not at present.

4) I have not had a perm since before treatment.

5) Others (Please describe in detail.)

Did you experience any problems or inconvenience when you started again? Describe in detail.

( )

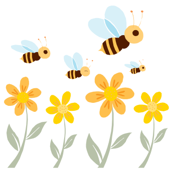

⑥ Hair dyeing (Circle the appropriate answer. Write numbers etc. in the blanks.)

Concerning the usage conditions.

1) Refrained from dyeing hair as compared to before treatment. → I used to dye my hair every ( ) months before treatment.

2) Started dyeing my hair again about ( ) months or ( ) years after the completion of treatment.

3) I dyed my hair before treatment, but I do not at present.

4) I have not dyed my hair since before treatment.

Did you experience any problems or inconvenience when you started again? Describe in detail.

( )

⑦ Did you make use of anything other than items ① to ⑥? (Circle one of the following.)

1) No 2) Yes

To patients who chose answer 2) to the above question. Describe in detail.

( )

⑧ What else did you want to use?

( )

2. To patients who used a wig.

① How many wigs did you buy? (Multiple answers allowed)

1) One
2) Two
3) Three
4) More than four
5) I bought a wig, but did not use it.
6) I did not buy one, but rented a wig.

② In which price range did you choose a wig? (Multiple answers allowed.) Answer by giving the price of one wig.

1) Less than 50,000 yen 2) more than 50,000 but less than 100,000 yen 3) 100,000 yen or more

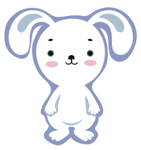

③ Did you have any problems choosing a wig? (Circle one of the following.)

1) No 2) Yes

This question is for patients who chose answer 2) to the above question. What was the problem? Please describe in detail.

( )

④ Have you had any problems using the wig? (Circle one of the following.)

1) No 2) Yes

This question is for patients who chose answer 2) to the above question. What problems did you have? Please describe in detail.

( )

⑤ How long did you use a wig after the completion of chemotherapy? (Circle one of the following. If you chose answer 1) to this question, please write numbers in the blanks.)

1) I used it for about ( ) years and ( ) months.

2) I am still using it.

07. Concerning information on scalp hair, eyebrows, eyelashes or nails and support that you actually received. If you received or experienced any form of support, please describe it.

1. Were you able to get any explanations or information about scalp hair, eyebrows, eyelashes or nails? (Circle one of the following.)

1) Yes → Please go to the next question ①.

2) No → Please go to question 2 (page 15).

① This question is for patients who answered “Yes” to the above question. Who/hat was the source of your information? Please check (tick) all of the boxes that apply.

□ Doctor① □ Nurse② □ Pharmacist③ □ Counselor at a counseling and support center④ □ Specialized staff handling hair loss⑤ □ Volunteer⑥ □ Family⑦ □ Friend⑧ □ Acquaintance⑨ □ Person with experience of cancer⑩ □ Internet⑪ □ Book⑫ □ Hair salon⑬ □ Others (Detail:)⑭

② Concerning information about scalp hair, was the explanation or information sufficient for you?
(Circle the appropriate answer.)

1) Sufficient 2) Almost sufficient 3) Partially sufficient<br>4) Not very sufficient 5) Not sufficient at all

This question is for patients who chose answer 1) or 2) to the above question. What information was useful?

Please describe in detail.

(   )

This question is for patients who chose answer 4) or 5) to the above question. Please describe the reason for the information being insufficient.

(   )

③ Concerning information about eyebrows, was the explanation or information sufficient for you?
(Circle the appropriate answer.)

1) Sufficient 2) Almost sufficient 3) Partially sufficient<br>4) Not very sufficient 5) Not sufficient at all

This question is for patients who chose answer 1) or 2) to the above question. What information was useful?

Please describe in detail.

(   )

This question is for patients who chose answer 4) or 5) to the above question. Please describe the reason for the information being insufficient.

(   )

④ Concerning information about eyelashes, was the explanation or information sufficient for you?
(Circle the appropriate answer.)

1) Sufficient 2) Almost sufficient 3) Partially sufficient<br>4) Not very sufficient 5) Not sufficient at all

This question is for patients who chose answer 1) or 2) to the above question. What information was useful?

Please describe in detail.

[ ]

This question is for patients who chose answer 4) or 5) to the above question. Please describe the reason for the information being insufficient.

[ ]

⑤ Concerning information about nails, was the explanation or information sufficient for you? (Circle the appropriate answer.)

1) Sufficient 2) Almost sufficient 3) Partially sufficient
4) Not so sufficient 5) Not sufficient at all

This question is for patients who chose answer 1) or 2) to the above question. What information was useful?

Please describe in detail.

[ ]

This question is for patients who chose answer 4) or 5) to the above question. Please describe the reason for the information being insufficient.

[ ]

⑥ When did you receive the information or support? (Multiple answers allowed)

1） Before treatment with anticancer agent(s)
2） During treatment with anticancer agent(s)
3） After the completion of treatment with anticancer agent(s)

2. If you have any ideas regarding information that you wanted after treatment, please provide them in the space below.

[ ]

3. If there is anything else you noticed about hair loss, please describe it to us.

[ ]

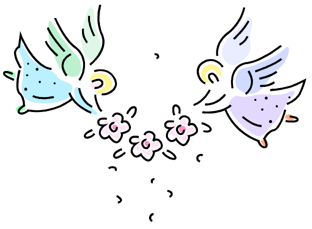

08. Finally, concerning your makeup at present and before treatment. Please circle the answer 1), 2) or 3) for each question.

A) Have you drawn eyebrows since before chemotherapy? • • • 1) Yes 2) No

B) Did you draw eyebrows during chemotherapy? • • • 1) Yes 2) No

C) Do you want to draw eyebrows regardless of chemotherapy? • • • 1) Yes 2) No

D) Did you know about art makeup (micro pigmentation) ? • • 1) Yes 2) No

E) Did you make use of art makeup (micro pigmentation) immediately before chemotherapy or during chemotherapy? • • • 1) Yes 2) No

<u>* To patients who answered Yes to question E).</u>

① Are you satisfied with the results of art makeup? • • 1) Very satisfied 2) Fair 3) Not satisfied

② Do you want to make use of art makeup regardless of chemotherapy? • • 1) Yes 2) No

F) Have you used false eyelashes during chemotherapy? • • 1) Yes 2) No

<u>* To patients who answered Yes to question F).</u>

① Were the false eyelashes useful? • • 1) Very useful 2) Fair 3) Not useful

② How often did you use the false eyelashes? • • 1) Occasionally 2) Sometimes 3) Almost every day

G) Did you take special care with your nails during chemotherapy? • • 1) Yes 2) No

<u>* To patients who answered Yes to question G).</u>

① Did you make use of manicures to hide your nail changes? • • • 1) Yes 2) No

② Did you make use of gel nails to protect your nails? • • • 1) Yes 2) No

③ Did you take special care to trim your nails? • • • 1) Yes 2) No

④ If you tried other techniques and strategies to manage your nails, please describe them in detail.

( )

H) What other forms of nail care did you want to try?

( )

End of the questionnaire.

Thank you for your cooperation.

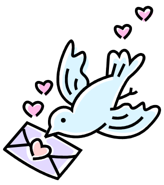

Supplement: S2 Fig — (PDF) [file pone.0208118.s003.pdf]
